# Formal leadership perceptions about the autonomy of Pharmacy: a SWOT analysis

**DOI:** 10.1016/j.rcsop.2024.100443

**Published:** 2024-04-10

**Authors:** Fernando de Castro Araújo-Neto, Aline Santana Dosea, Francielly Lima da Fonseca, Thaís Maria Tavares, Déborah Mônica Machado Pimentel, Alessandra Rezende Mesquita, Divaldo Pereira de Lyra-Jr.

**Affiliations:** a Graduate Program in Pharmaceutical Sciences. Laboratory of Teaching and Research in Social Pharmacy (LEPFS), Federal University of Sergipe, São Cristóvão, Sergipe, Brazil; b Health Sciences Graduate Program. Laboratory of Teaching and Research in Social Pharmacy (LEPFS), Federal University of Sergipe, São Cristóvão, Sergipe, Brazil; c Departament of Medicine, Hospital Universitary of Sergipe – Federal University of Sergipe, Aracaju, Sergipe, Brazil; d Laboratory of Teaching and Research in Social Pharmacy (LEPFS), Federal University of Sergipe, São Cristóvão, Sergipe, Brazil

**Keywords:** Pharmacy, Professionalism, Content analysis, Environmental analysis, SWOT analysis, Autonomy

## Abstract

Introduction: Autonomy is considered a vital principle of professionalism. In recent years, despite important advances, the Pharmacy and pharmacists' autonomy has been questioned due to conflicts that jeopardize the consolidation of this profession in the division of work in health. Objective: to understand the construct of autonomy based on perceptions of formal leaders associated with professional organizations. Methods: A qualitative study was conducted through interviews with key informants. The data obtained were submitted to content analysis. Results: Perceptions about the autonomy in pharmaceutical practice were categorized according to strengths, weaknesses, opportunities, and threats to this construct. Conclusion: The findings allowed us to understand the autonomy of pharmaceutical practice in Brazil, generate hypotheses about the future of Pharmacy, and build strategies to maintain its occupational status.

## Graphical abstract

**Figure:**
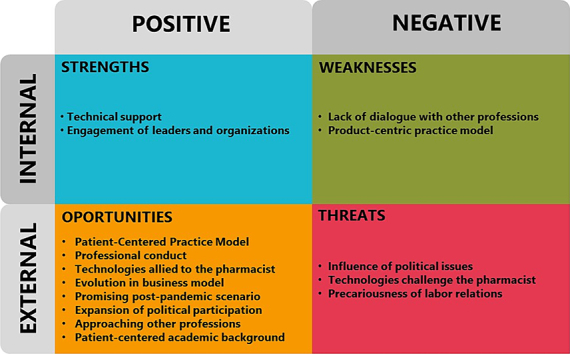

## 1 Introduction

With the disenchantment of the world and bureaucratization of post-agrarian life, professions emerged to formalize the division of work and produce goods and services based on social needs.^1^, ^2^, ^3^ In the 20th century, professionalism arose as a sociological current to understand the occupational strategies of professions around State and market demands.^4^^,^^5^ Over time, autonomy, which is the ability of the professional to control their own work without interference from any layperson or other corporations, has become a cornerstone of professionalism.^6^, ^7^, ^8^

From the 1960s onwards, representative organizations and their formal leaderships have guided the “reprofessionalization” of Pharmacy to a patient-centered care, regaining prestige through the provision of medicine-related services.^9^, ^10^, ^11^ In other words, the social role of Pharmacy has been changing in different levels such as attitudinal, specially related to the behavior of pharmacists; political, related to the profession's capacity for political articulation; technical, related to the ability to deliver clinical services; and administrative, related to the dynamism of administrative and management processes that has emerged in this new era.^12^, ^13^, ^14^, ^15^ Despite all that, some gaps caused by internal and external factors can threat the autonomy of pharmacists, such as self-deprecation and employment dependent on large drug retail corporations.^6^

Therefore, professional organizations of Pharmacy have been putting effort to encourage pharmacists to develop their autonomy before the challenges imposed in health services.^6^^,^^16^, ^17^, ^18^ In this context, formal leaderships of the profession have a critical role due to their representative nature in favor of the Pharmacy and pharmacists' interests in these scenarios.^7^^,^^17^^,^^18^ Besides that, they are the stakeholders responsible for designing strategies that link social demands to the Pharmacy interest of consolidating itself as a clinical profession.^6^^,^^17^, ^18^, ^19^, ^20^

Thus, the objective of this study was to understand the construct of autonomy in the pharmaceutical practice from the perceptions of formal leaders associated with professional pharmaceutical organizations.

## 2 Methods

### 2.1 Study design

A qualitative study was developed between July 2020 and February 2021. Data were obtained from interviews with individuals that hold formal leadership positions in pharmaceutical organizations and associations in Brazil. Formal leaders must represent the interests of the profession and professionals to society.^21^, ^22^, ^23^ They are responsible for planning and executing tasks and legal prerogatives to provide services, granting the influence and appreciation of the profession. Thereby, they safeguard the autonomy of professionals, which is one of the essential principles of professionalism.^6^^,^^18^^,^^24^ In Brazil, pharmacists are represented by the Federal Pharmacy Council, which is responsible for regulating and supervising professional practice.^6^^,^^18^ Associations and unions are responsible for promoting the appreciation of pharmaceutical work in society, through continuing education and salary policies.^6^^,^^18^ Currently, the associations responsible for these activities are the National Federation of Pharmacists, the biggest union representation of Brazilian pharmacists, besides other associations from different areas of Pharmacy that aim to promote continuing education and the valuation of the pharmacist work in multiple scenarios. These entities also influence the formal education of Pharmacists, which last approximately five years. Likewise, they influence the proposal, execution, and evaluation of public policies for the work process of pharmacists in the private sector and in the Brazilian National Health System (SUS).^6^^,^^16^, ^17^, ^18^^,^^25^^,^^26^ Thereunto, this study followed the recommendations proposed by the Consolidated Criteria for Reporting Qualitative Research (COREQ).^27^

### 2.2 Study participants

The study sample consisted of pharmacists that are formal leaders associated with the management or consultancy of organizations, associations, unions, and entities of pharmaceutical practices in Brazil. The interviewees were selected using two techniques as follows: intentional sampling, which means that the researchers conveniently selected participants; and “snowball” sampling, that is, the interviewees suggested other potential participants.^6^^,^^28^^,^^29^ Nevertheless, the interviewees should occupy a leadership position at any organization from those consulted previously. During the initial approach, carried out by e-mail, the interviewees became aware of the objective of the study and attested their awareness by signing an informed consent form.

### 2.3 Script development

The researchers developed a script (FCAN, ASD, FLF) based on the neoWeberian perspective of the study of professions.^1^^,^^2^^,^^7^ In this perspective, professions are analyzed considering changes in the evolution of social needs, as well as the autonomy delegated to professionals to provide goods and services to consumers.^1^, ^2^, ^3^^,^^7^ This script was developed during brainstorming sessions with the researchers (FCAN, ASD, FLF, ARM, DMMP), and was reviewed by the senior researcher (DPLJ) to improve the semantic, conceptual, and theoretical issues that would enable interviewees to understand the questions. The objective was to approximate the interviewer (FCAN, male, MSc) and the interviewees to discuss relevant topics and allow that other potentially relevant topics were considered during the interviews.^28^^,^^30^

Based on this premise, the following guiding questions were added to the script: “How do you see pharmacists' autonomy in playing their (social) role?” “What is your perception of pharmaceutical services from the perspective of the society's needs?” “What are your expectations for the future of Pharmacy?”

### 2.4 Interviews

Due to the sanitary measures of social isolation imposed by the COVID-19 pandemic in 2020, interviews were conducted using the Skype™ platform. The interviewer (FCAN, male, MSc) was a researcher in professionalism who had no personal bonds with the interviewees. The role of the interviewer was to stimulate answers from the interviewees but he did not interfere in the content of the answers or statements given during interviews.^27^^,^^31^ In addition to record the content in audio and video, the interviewer/researcher used a logbook during the process so that potential impressions, issues to be explored in later interviews, mannerisms, and non-verbal communication elements could be explored in later analysis.^32^

### 2.5 Data saturation

During the interviews, procedures inherent to data saturation were adopted when the interviewer noticed that a new analytical phenomena did not arise or when there was repetition of themes and opinions expressed in previous interviews.^33^ After each interview, the researchers (FCAN, ASD, FLF) analyzed the data obtained to observe emerging themes and avoid redundancies in the topics covered by the interviewees, as well as statements that were not align with the analytical phenomenon of the research. The number of interviews was another indicator used to define the data saturation. Currently, the literature considers that an ideal number of interviews to reach the data saturation is around nine and twenty-four interviews.^6^^,^^29^^,^^34^

### 2.6 Reflexivity

Reflexivity in qualitative research involves recognizing and comprehending the prior influences of researchers during the development of the study. However, it emphasizes that these influences should not be predominant during the execution of the project to prevent bias.^35^^,^^36^ Thus, all these elements were thoroughly discussed among the researchers so that they would not influence the drafting of the script, the choice of interviewees, the interviews, and the analysis of the data.

### 2.7 Data analysis

Two researchers (FCAN, TMAT) completely transcribed all the content recorded in audio and video. Three researchers (ASD, FCAN, FLF) individually and inductively analyzed the transcriptions using the technique proposed by Bardin.^37^ After that, they came to a consensus through meetings using the software ATLAS.ti.^38^ At first, the group (ASD, FCAN, FLF) conducted a pre-analysis of the material which consisted in floating readings to organize the content.^39^ The video records were also checked to capture some non-verbal communication elements of the interviewees which may somehow influence the data analyzes.

Subsequently, three researchers (ASD, FCAN, FLF) analyzed the material independently by reading it and reading again to develop a selection of excerpts and units of meaning. In the sequence, the coding were generated inductively, that is, they emerged directly from the analyzed data and from the researchers' search for convergences and divergences among them, at the most diverse levels of interpretation and abstraction.^39^^,^^40^

Next, when the generated codes were deemed sufficient for the description of the phenomenon (professional autonomy in the professionalism context) and the generation of hypotheses that could be discussed, the researchers shared the results of their codifications in meetings. The group agreed upon the themes and codes related to the research questions allowing a posterior discussion of the analyzed content. The codes were then reviewed and possible differences in wording were resolved together with a senior researcher.^27^^,^^28^

### 2.8 SWOT Analysis (Environmental Analysis)

After inductive coding was generated during the analysis of the content of interviews, the codes were submitted to an empirical analysis by the researchers (FCAN, ASD, FLF, DPLJ) and classified according to the SWOT matrix. This procedure, derived from theories of environmental analysis, allowing to categorical organize the generated codes according to internal factors represented by strengths (S) and weaknesses (W), and external factors symbolized by opportunities (O) and threats (T) to the autonomy of pharmaceutical practices.^41^^,^^42^

### 2.9 Ethical aspects

This study was approved by the Research Ethics Committee of the Federal University of Sergipe - Brazil (protocol number: 4102149).

## 3 Results

### 3.1 Characterization of the participants

Seventeen pharmacists participated in this study and most of them were female (10; 58.8%). The average age was 54 years, and eight (47.05%) respondents had PhD. In this study, all interviewees held management or consulting positions in the regulatory authority of Pharmacy in Brazil or in associations that represent the interests of pharmacists.

In addition, the areas of activity reported were clinical pharmacy, hospital pharmacy management, pharmacy education, and community pharmacies. Each interview lasted an average of 43 min and generated approximately 780 min of recorded audio and video content.

**Fig. 1:**
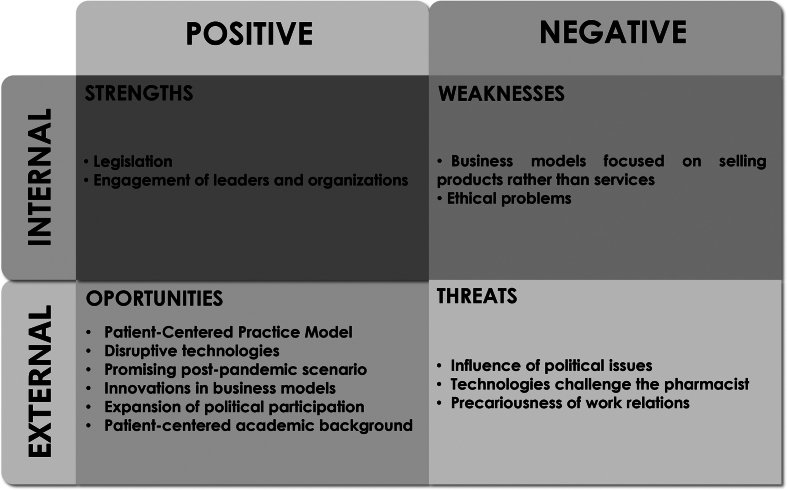
Characterization of pharmaceutical autonomy: SWOT approach

Based on inductive coding, the themes were categorized into positive, negative, internal, and external factors that may influence the autonomy component in the context of pharmaceutical professionalism. Fig. 1 shows the internal and external factors of Pharmacy, such as strengths, weaknesses, opportunities, and threats. Extracts from the interviewees' speeches are identified by codes.

### 3.2 Characterization of pharmaceutical autonomy: SWOT approach

#### 3.2.1 Internal factors

##### 3.2.1.1 Strengths

I1 : “Before, there were many lawsuits by Pharmacy technicians or practitioners who claimed the technical responsibility of community pharmacies. Following the new legislation in 2014, it was decided that the pharmacist was the only technician responsible for the pharmacy.”

In general, the interviewees stated that the legislation that provides technical support to professional activity strengthens the autonomy of pharmacists. Some federal laws have been quoted as fundamental milestones for Pharmacy in Brazil and have contributed to the maintenance of the occupational status of the profession. The awareness and engagement of leaders in strategic issues to pharmaceutical practice can provide autonomy to pharmacists as these practices bring them closer to society while they work to deliver services in health establishments. According to the interviewees, the strength of councils and scientific societies is fundamental to regulate the professional practice, ethical guidance, work processes, and indicators of quality.

##### 3.2.1.2 Weaknesses

I2 : “In the pandemic, unfortunately, some pharmacists showed their worst. They showed that we pharmacists are salespeople, when they sold ivermectin, which is a medicine without any technical-scientific evidence, hydroxychloroquine…”.

In this study, the interviewees reported that business models that aim the sale of products rather than services, as seen in community pharmacies, weakens the autonomy of pharmacists. To some interviewees, in these scenarios, there is an extremely exploration of the work force, besides, pharmacists are forced to sale medicines and other products without caring about the patient safety.

I3 : “I had colleagues (pharmacists) who reported that the manager (of the community pharmacy) did not authorize informing the patient about the risks of medications. Pharmacists couldn't tell the risks associated with the use of hydroxychloroquine, because the manager said the patient would not purchase the product if he was informed of the risks".

The interviewees believed that there were ethical problems during the COVID-19 pandemic, as some community pharmacies and hospitals violated the technical autonomy of pharmacists and forced them to dispense or distribute medicines or supplements without scientific evidence in the context of the prevention or treatment of the new coronavirus. The negative influence of the labor market on the distribution of medicines or the sale of health products rather than the development of pharmaceutical services was also highlighted.

### 3.3 External factors

#### 3.3.1 Opportunities

I4 : “I think what is missing is for society to recognize the profession. People know the teacher and the nurse, and it is necessary to recognize the pharmacist.”

As reported by the informants, Pharmacy as a profession should take advantage of opportunities related to the social needs to adopt patient-centered models of practice and service provision. Therefore, it is necessary to present a proactive vision, good personal conduct, attitudes, behaviors, and position to health professionals. During the pandemic, the lack of information about COVID-19 and its treatments created the need for remote pharmaceutical care.

I5 : “Just as the doctor has his office, the pharmacist can have his virtual clinic, he can provide services through telepharmacy.”

Thus, telepharmacy, as a disruptive technology that brought professionals and patients closer to health prevention, promotion, and rehabilitation. Innovations in business models evolve along with entrepreneurship before the detachment of pharmacists from private companies that explore their workforce. Therefore, the informants pointed out that this is an opportunity for Pharmacy to approach its work processes to those of physicians, for example.

I6 : “We are in community pharmacies to protect people and not to sell products. It is not our main function to sell products. We are inside pharmacies to protect people against the irrational use of medicines. I need to guarantee access, I need to guarantee information and I need to guarantee the protection of these people.”

As the pharmaceutical practice is being redirected towards patient-centered services, the informants indicated that the global scenario after the COVID-19 is an opportunity for the consolidation of Pharmacy which must be recognized as an ally in the fight against the pandemic. This recognition is aligned with the political participation of Pharmacy and pharmacists in elective positions, political articulations, and entities that represent these professionals in the country. This effort is an opportunity to make Pharmacy more representative and with greater political power, contributing to the elaboration and execution of health policies besides generating greater visibility in society.

According to the interviewees, bringing pharmacists closer to other health professionals as physicians and nurses by developing services together, is fundamental for the survival of the profession. In this sense, it was highlighted that the academic training is a strategic component, and its focus should be in patient healthcare rather than the product, yet not losing its technological bias. Therefore, in the teaching context, opportunities for Pharmacy depend on the evolution of the individual-centered model, which steps away from the biomedical logic, in which the foremost focus is on the disease, and embrace that psycho-social aspects are also determining in the health-disease process.

#### 3.3.2 Threats

I7 : “Autonomy, in fact, is a utopia, right now.”

According to the interviewees, the technical autonomy of Pharmacy is fluid and threatened. The lack of autonomy is considered a threat to the profession and is influenced by issues of political interest articulated between consumer market strengths and legislative proposals to reduce the pharmacist participation in the provision of services. An example given by the interviewees consisted of a bill that aimed to eliminate the mandatory presence of pharmacists in community pharmacies, that is, the autonomy that should be an irrevocable privilege tends to be limited by external strengths.

For the leaders interviewed, autonomy can be reduced when the pharmacist submits to the expectations of companies that buy their workforce. According to the interviewees, in environments such as hospitals, the division of work is not hierarchical, and the pharmacist is “free” to work in a team. In community pharmacies, the current business model focuses on the sale of products rather than delivering services and requires that pharmacists deal with drug trades instead of the patient care.

I8 : “Pharmacists are not class conscious. They do not recognize themselves as a working class and they do not understand themselves as a working class.”

As one of the informants described, there is a concern about the proletarianization of Pharmacy based on phenomena such as deregulation, precariousness of work relations, and low salary profitability. At the same time, although entrepreneurship is seen as an opportunity, it is characterized as a threat to profession achievements as a working class. According to the informants, despite moral recognition was an essential activity during the COVID-19 pandemic, the appreciation of the pharmaceutical work process and its remuneration are still insignificant and affects long-term survival.

I8 : “In this world we live in, it is not possible to imagine a digital profession.”

Another issue that arose was the use of technology within the profession. For the interviewees, these advances can surpass the pharmacist workforce, posing as a threat to the profession. In a future that appears “nebulous”, one fear hangs over pharmacists' heads: that they may become obsolete for not being able to keep up the technological innovations or incorporate these devices in their practice models.

I9 : “The pharmaceutical profession must continue its development process without losing its guidance service.”

Interviewees believe that the excessive link between the pharmacist and the medicine to the detriment of patients is associated with a technological dilemma. Thus, linking the profession only to the product can be risky if its model of practice is not reframed.

## 4 Discussion

### 4.1 Strengths

This study analyzed and categorized the perception of formal leaders of pharmaceutical practice in Brazil about the autonomy of Pharmacy in accordance with the SWOT matrix. Regarding the strengths of the profession, it was especially highlighted the recent regulatory frameworks that contributed to the establishment of Pharmacy as a clinical profession.^3^^,^^7^^,^^43^

Corroborating the perceptions of those interviewed, these issues are facilitated, especially through the articulation of professional councils, organizations, and associations together with society and the State. In Brazil, the industrialization of medicines moved pharmacists away from community pharmacies and hospitals by getting them to carry out technical tasks or assisting Medicine.^9^^,^^44^ Consequently, the profession has been intimidated by legal mechanisms that aim to extinguish the responsibility of pharmacists in community pharmacies.^45^^,^^46^

Despite the approval of a new legislation that reinforces the pharmacist's authority as a health professional, in Brazil, there are still some contradictions as a law from the 1970s that was not revoked and addresses the technical responsibility in the community pharmacy without mentioning the pharmacist.^46^, ^47^, ^48^ Some informants reported that this device in the 1970s opened the door to “practitioners” who, even without academic training, acted as technical officers and demanded recognition of their services in justice. In other words, the closer the professions and their organizations are to those who own the means of producing goods and services, the greater they guarantee their technical autonomy and occupational control.^49^

### 4.2 Weaknesses

Despite the interest of bureaucratic bodies in requesting the service of occupational groups, as highlighted by some interviewees, without the engagement of professionals and their leaders, the process of consolidation of the profession is inefficient. In this constant cold war, the right to control its own work was granted to professions that produces enough knowledge, ideologies, and ethical dictates to be seen as indispensable to social control.^3^^,^^4^^,^^7^^,^^38^

Under these conditions, other factors sound like internal criticisms and weaknesses to the Pharmacy's occupational project. Historically, pharmacists enjoyed prestige in society prior to their professionalization.^50^ In certain sites, where the state does not exercise control over health policies or in economic models where the private sector experience greater freedom, pharmacists manage to control the division of work in health and the pharmacy is the reference for urgencies or emergencies in that region.^2^^,^^44^

However, these weaknesses are sometimes associated with the fear of taking new responsibilities and the difficulty of providing the services physicians and patients expect.^51^ Yet, although legal provisions guarantee technical autonomy, the hierarchy still remains, for example, when the number of pharmaceutical interventions accepted by physicians is used as an indicator.^52^

As the interviewees said, the fear of taking new responsibilities is implicit in the training model of pharmacists. According to the literature, the autonomy of pharmacists to control their own work depends on the acquired knowledge and the way it is transmitted to their treinees.^1^^,^^3^^,^^4^ For interviewees, communication, for example, is a curricular component that has not been fully elucidated and should be considered an essential element. Thus, transmitting an exclusive body of knowledge is as important as teaching skills, which allow pharmacists to interact with patients and other professionals.^53^^,^^54^

In Pharmacy, the abandonment of the processes related to the research, development, and distribution of medicines would probably cause more damage than harm, as some interviewees defended. Thus, the alternative is to approximate ideology and teaching, with the consolidation of models that meet the demands of the public health system, as the integration of the medicine access management and monitoring in health services of the private sector.^26^

### 4.3 Opportunities

According to the data analysis obtained from the interviews, external factors can be interpreted as opportunities for the pharmaceutical practice and its autonomy. In addition, they can be strategic for the occupational survival of health professionals, such as the adoption of patient-centered practice models. After realizing the decline of their influence on society, Medicine and Nursing, for example, adopted skills to bring professionals closer to their patients.^55^, ^56^, ^57^

According to the literature, the theoretical basis of an exclusive knowledge that should be inaccessible to laypeople and other professionals is fundamental to build the autonomy of a profession.^2^^,^^38^ In the 20th century, based on the criticism that health professions are altruistic instruments that aim to serve society, physicians, nurses, and pharmacists sought to understand the biopsychosocial aspects of patients, which led collaborative and interprofessional practices to gain space in the context of health care.^55^

Therefore, the current business model focused on medicines and linked to the sale of the workforce of pharmacists in private community pharmacies should be replaced by the ideology of the profession, incorporating independent patient care services even in public health systems, encouraging and confirming the prestige, power and autonomy of the profession.^58^^,^^59^

As in other professions that incorporate technological advances in favor of their models of practice, Pharmacy needs to interpret automation, artificial intelligence, big data, wearable devices, and telepharmacy as an opportunity to build bridges to users of its services.^19^ From the perspective of telepharmacy, respondents mentioned that pharmacists could meet the demands of patients residing in remote locations providing health services. Therefore, the technology is used as a strategic alternative for pharmacists and their organizations, whether in the regulatory context or in education.^60^^,^^61^

The use of technologies that bring patients and healthcare professionals together was deepened during the COVID-19 pandemic, raising the occupational status of pharmacists in different parts of the world.^62^^,^^63^ Still, the fake news infodemic about COVID-19 medicines, the race to develop vaccines, and policies in public and private health systems are noteworthy.^64^^,^^65^ Once again, the approximation of bureaucratic bodies is fundamental to the autonomy of Pharmacy through political participation and collective efforts to stand out before patients and health systems.^43^^,^^66^^,^^67^

### 4.4 Threats

The interviewees considered the influence of political issues as a weakness that must be overcome by the profession. It is essential that professions define their attributions and jurisdictions among themselves, respect their complementarities without overlapping practices, and disclose and clarify their different roles to society.^68^^,^^69^ Searching for autonomy, professions represented by their leaders produce informal competition among themselves, alerting society to exclusive services or services shared with other corporations.^3^^,^^8^

The evolution of professional practice models should be noted. Some interviewees believe that technologies can play a dual role of facilitating clinical services and threating the sustainability of the profession. In addition, the literature describes situations in which pharmacists are involved with mechanical functions that may be replaceable by machines or professionals with less expertise.^16^^,^^70^ Incorporating these innovations can make work relations precarious and questions the liberal condition of occupations and their functionalist way.^71^ Thus, it is necessary to discuss the dilemmas between the efficiency of technologies in the provision of services and the threat to pharmacists' occupational survival.^19^^,^^72^

This study has some limitations. The COVID-19 pandemic did not allow that interviews were conduct face-to-face, although the literature highlights that remote interviews are an acceptable resource in such conditions. Besides, no feedback was given to participants about the findings of the study nor transcripts were returned. However, as far as we know, this is the first study that explores the perception of formal leaders of Pharmacy in Brazil concerning the autonomy of the profession.

## 5 Conclusion

In this study, pharmaceutical practice leaders in Brazil reported their perceptions of the autonomy of their profession. Despite the strengths related to legislation, engagement of pharmacists, and many opportunities for the solid development of their practice models, there are weaknesses and threats that challenge their sustainability. Therefore, the results suggest that autonomy is a complex and abstract concept that is in a continuous construction.

Therefore, pharmacists and their formal leaderships must articulate strategies that guarantee the Pharmacy survival specially through a universal definition of which are the priorities of Pharmacy before the current challenges and dilemmas. Also, it is necessary to establish and consolidate care services for the patient, family, and community.

## Funding

This study was financed in part by the Coordenação de Aperfeiçoamento de Pessoal de Nível Superior – Brasil (CAPES) – Finance Code 001.

## Author contributions

**Fernando de Castro Araujo Neto.**

Conceptualization, Methodology, Investigation, Formal analysis, Data Curation, Writing - Review & Editing.

**Aline Santana Dosea.**

Conceptualization, Methodology, Investigation, Formal analysis, Data Curation, Writing - Review & Editing.

**Francielly Lima da Fonseca.**

Conceptualization, Methodology, Investigation, Formal analysis, Data Curation, Writing - Review & Editing.

**Thais Maria Araújo Tavares.**

Conceptualization, Methodology, Investigation, Formal analysis, Data Curation, Writing - Review & Editing.

**Deborah Monica Machado Pimentel.**

Conceptualization, Methodology, Investigation, Formal analysis, Data Curation, Supervision.

**Alessandra Rezende Mesquita.**

Conceptualization, Methodology, Investigation, Formal analysis, Data Curation, Supervision.

**Divaldo Pereira de Lyra Junior.**

Conceptualization, Methodology, Investigation, Formal analysis, Data Curation, Supervision, Project administration.

## CRediT authorship contribution statement

**Fernando de Castro Araújo-Neto:** Writing – review & editing, Writing – original draft, Visualization, Methodology, Investigation, Formal analysis, Data curation, Conceptualization. **Aline Santana Dosea:** Writing – review & editing, Writing – original draft, Methodology, Investigation, Formal analysis, Data curation, Conceptualization. **Francielly Lima da Fonseca:** Writing – review & editing, Writing – original draft, Methodology, Investigation, Formal analysis, Data curation, Conceptualization. **Thaís Maria Tavares:** Writing – original draft, Methodology, Data curation, Conceptualization. **Déborah Mônica Machado Pimentel:** Writing – review & editing, Supervision, Methodology, Conceptualization. **Alessandra Rezende Mesquita:** Writing – review & editing, Writing – original draft, Visualization, Data curation, Conceptualization.**Divaldo Pereira de Lyra-Jr:** Supervision, review, writing and project administration.

## Declaration of competing interest

The authors declare that they have no known competing financial interests or personal relationships that could have appeared to influence the work reported in this paper.
